# 847. A Qualitative Exploration of New and Existing HIV PrEP Modalities Among Men Who Have Sex with Men in Philadelphia

**DOI:** 10.1093/ofid/ofab466.1042

**Published:** 2021-12-04

**Authors:** Nguyen K Tran, Omar Martinez, Neal Goldstein, Ayden Scheim, Seth Welles

**Affiliations:** 1 Drexel University Dornsife School of Public Health, Philadelphia, PA; 2 Temple University, Philadelphia, PA; 3 Drexel University, Philadelphia, PA

## Abstract

**Background:**

Daily oral pre-exposure prophylaxis (PrEP) is highly effective at preventing HIV when used as directed among men who have sex with men (MSM). However, challenges with uptake and adherence to daily oral PrEP have prompted the development of new modes of administration, including a long-acting, intramuscular injectable. We sought to explore the treatment characteristics that may influence the willingness and uptake of long-acting injectable PrEP as opposed to the daily pills among a racially diverse sample of MSM.

**Methods:**

Between January and May 2021, we actively recruited 28 HIV-negative MSM (8 Black, 10 Latinx, 10 White) who lived in Philadelphia, PA during the past 12 months using social networking sites (e.g., Facebook and Instagram) and a community listserv. Qualitative data collection used a hybrid approach in which 4 focus groups and 10 semi-structured interviews were conducted virtually. Focus groups were kept racially and ethnically homogenous to identify differences in emerging themes related to PrEP willingness and preferences for specific prevention modalities.

**Results:**

Participants discussed differing levels of interest and willingness to use long-acting injectable PrEP as opposed to the daily pills. The main perceived facilitator for injectable PrEP included convenience of use such as having fewer concerns with adhering to daily pills. Perceived barriers to injectable PrEP included (1) a dislike of needles as well as (2) concerns of potential side effects and (3) lower treatment efficacy (i.e., whether it will be as effective as the daily pills). While Black and Latinx MSM reported experiences of racism and discrimination within the healthcare system, they also reported greater willingness to consider intramuscular injectables if their healthcare providers would provide in-depth information about the risks and benefits of this new modality.

**Conclusion:**

Our findings provide important guidance for the development and promotion of future strategies to enhance the uptake of long-acting injectable PrEP to address the HIV epidemic among MSM. Primary care providers should play a key role in ameliorating concerns related to hesitancy towards injectable PrEP, including emphasizing ease of dosing, effectiveness, and safety of long-acting PrEP to prevent infection.

**Disclosures:**

**All Authors**: No reported disclosures

